# Click-to-Chelate: Development of Technetium and Rhenium-Tricarbonyl Labeled Radiopharmaceuticals

**DOI:** 10.3390/molecules18033206

**Published:** 2013-03-12

**Authors:** Christiane A. Kluba, Thomas L. Mindt

**Affiliations:** Division of Radiopharmaceutical Chemistry, Department of Radiology and Nuclear Medicine, University of Basel Hospital, Petersgraben 4, 4031 Basel; Switzerland; E-Mail: Christiane.Kluba@usb.ch

**Keywords:** CuAAC, Click-to-Chelate, radiolabeling, radiopharmaceuticals, ^99m^Tc-tricarbonyl, ^186/188^Re-tricarbonyl

## Abstract

The Click-to-Chelate approach is a highly efficient strategy for the radiolabeling of molecules of medicinal interest with technetium and rhenium-tricarbonyl cores. Reaction of azide-functionalized molecules with alkyne prochelators by the Cu(I)-catalyzed azide-alkyne cycloaddition (CuAAC; click reaction) enables the simultaneous synthesis and conjugation of tridentate chelating systems for the stable complexation of the radiometals. In many cases, the functionalization of (bio)molecules with the ligand system and radiolabeling can be achieved by convenient one-pot procedures. Since its first report in 2006, Click-to-Chelate has been applied to the development of numerous novel radiotracers with promising potential for translation into the clinic. This review summarizes the use of the Click-to-Chelate approach in radiopharmaceutical sciences and provides a perspective for future applications.

## 1. Introduction

Even though new generator-based radionuclides (e.g., ^68^Ga) are emerging [1], technetium 99m remains the workhorse of nuclear medicine due to its convenient availability via ^99m^Mo/^99m^Tc generators, reasonable cost, and ideal decay characteristics (*t*_1/2_ = 6.02 h, 140 keV, 89% abundance) for Single Photon Emission Computed Tomography (SPECT). Approximately 80% of all routine nuclear imaging applications are based on ^99m^Tc, which translates into estimated 50 million doses of diagnostic radiopharmaceuticals annually worldwide, or more than 50,000 applications per day in the US alone [2,3]. In addition, the availability of *β*-particle-emitting rhenium 186/188 as group 7 transition metal congeners of ^99m^Tc with similar chemical properties provides a “matched pair” constellation of radionuclides for both diagnostic and therapeutic applications. The possibility to radiolabel molecules of medicinal interest with diagnostic or therapeutic radionuclides by the same chemistry approach has recently received considerable interest in the field of nuclear medicine for the development of theranostic agents [4].

**Scheme 1:**
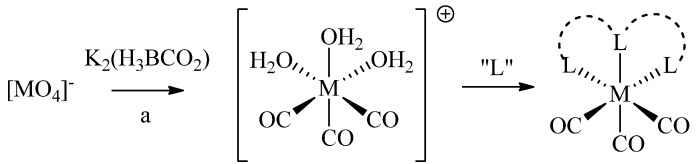
Synthesis of [M(CO)_3_]^+^ from permetallates and subsequent complexation with ligands (L). (a) For M = ^99m^Tc: (i) IsoLink^TM^ kit (containing K_2_[H_3_BCO_2_]), saline, 100 °C, 20 min; for M = ^188^Re: (i) SnCl_2_, gluconate, H_3_PO_4_; (ii) K_2_[H_3_BCO_2_].

Because of the diverse redox chemistry of technetium and rhenium, a wealth of complexes differing in coordination numbers and geometries has been reported. Here, we focus on the technetium and rhenium-tricarbonyl cores ([M(H_2_O)_3_(CO)_3_]^+^, or short [M(CO)_3_]^+^; M = ^99m^Tc(I), ^186/188/nat^Re(I)). ^99m^Tc- and ^186/188^Re-tricarbonyl can be prepared by reduction of the corresponding permetallates ([M(VII)O_4_]^−^) using K_2_[H_3_BCO_2_] (e.g., by the commercial IsoLink^TM^ kit; Scheme 1). [M(CO)_3_]^+^ contains three tightly coordinated CO ligands and three water molecules, the latter of which can be readily replaced by mono-, bi-, and tridentate ligand systems, or a combination thereof. Complexes of [M(CO)_3_]^+^ exhibit a low spin d^6^ configuration, which renders the metal inert and hence, provides complexes with high *in vivo* stability essential for medical applications [5]. In addition, octahedral complexes of [M(CO)_3_]^+^ are generally smaller than octahedral or square-pyramidal complexes of the corresponding metals in higher oxidation states (e.g., M(V)-oxo cores) and are therefore considered less likely to impact important characteristics of (bio)molecules to which they are conjugated to.

**Figure 1:**
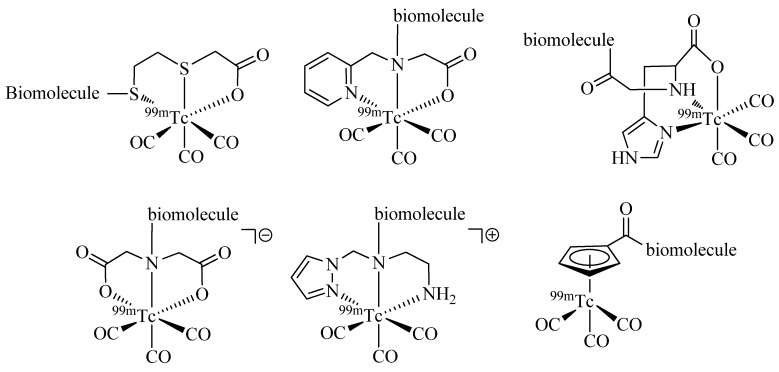
Representative examples of bifunctional chelating agents (BFCAs) for the complexation of the [^99m^Tc(CO)_3_]^+^ core and conjugation to (bio)molecules.

Since the first report of the ^99m^Tc-tricarbonyl core [^99m^Tc(H_2_O)_3_(CO)_3_]^+^ in 1998 by Alberto and Schibli *et al*. [6] numerous ligand systems and their coordination with [M(CO)_3_]^+^ have been investigated. In particular, tridentate chelators containing *N*-, *S*-, or *O*-donors have been shown to provide well-defined and stable organometallic complexes, which are suitable for application *in vivo* (Figure 1) [7,8] Also, functionalized η^5^-ligand cyclopentadienide as well as carboranes [9] have been used for the stable complexation of the tricarbonyl core. A thorough discussion of the literature on ligand systems reported for the complexation of [M(CO)_3_]^+^ is beyond the scope of this article and instead, it is referred to some excellent reviews on the general topic of ^99m^Tc chelation chemistry (see e.g., [10]).

Radiolabeling of molecules of medicinal interest with [M(CO)_3_]^+^ is usually achieved by a post-labeling approach using bifunctional chelating agents (BFCAs). As the name implies, BFCAs enable both the covalent linkage to a (bio)molecule and the coordination of the radiometal. For many of the ligand systems reported for [M(CO)_3_]^+^, appropriately functionalized derivatives for conjugation to (bio)molecules via different functional groups (e.g., amines, carboxylates, thiols) have been reported [10]. However, the multifunctional character of both the ligand system and (in most cases) the (bio)molecule of interest can make a selective conjugation in solution a difficult endeavor. To overcome these problems, protective group strategies are often employed; though, such approaches usually result in multi-step reaction sequences, which can significantly lower the overall efficiency of the labeling procedure.

Despite the success of the metal tricarbonyl cores in the field of radiopharmaceutical sciences, there is still a need for novel and innovative strategies for (bio)conjugation techniques and radiolabeling procedures in order to expedite the development of radiotracers based on [M(CO)_3_]^+^. In the following, this review will focus on such a recently developed approach that utilizes click chemistry in this context.

## 2. Click Chemistry in Radiopharmaceutical Sciences

Click chemistry, a term minted by Sharpless [11] *et al*. in 2001, is an attempt to define the characteristics of a perfect (or nearly perfect) chemical reaction that generates structurally diverse new substances by joining small building blocks in a quick, selective, efficient, and reliable manner. The prime example of a click reaction within the click chemistry concept is the copper (Cu(I))-catalyzed azide-alkyne cycloaddition (CuAAC), reported at the same time by the groups of Sharpless and Meldal in 2002 [12,13]. The CuAAC proceeds efficiently and selectively under mild reaction conditions (e.g., in water at room temperature) in the presence of other functional groups, and provides a stable 1,4-disubstituted 1,2,3-triazole heterocyclic linkage between the reaction partners. Not surprisingly, the attractive features of this click reaction have inspired numerous applications across different scientific disciplines [14,15,16], including examples of the development of radiotracers.

The first examples on the use of the CuAAC in radiopharmaceutical sciences were published in 2006. Kolb *et al*. [17] and Marik *et al*. [18] described the click reaction of [18F]fluorinated alkyne derivatives with azide acceptors, such as azido thymidine (AZT) and azide-functionalized peptides. These applications employ a two-step prosthetic group approach, which enables the efficient and selective ^18^F radiolabeling of molecules in aqueous media. For an overview on the CuAAC-mediated radiolabeling of molecules with ^18^F- (and ^11^C-) radionuclides, it is herein referred to some of the several recent review articles on the topic [19,20].

Also in 2006, Mindt and Schibli *et al*. reported the first example of an application of the CuAAC to metallic radionuclides [21]. In their approach, the click reaction is employed to the synthesis of tridentate ligand systems for the stable complexation of [^99m^Tc(CO)_3_]^+^ while conjugating them simultaneously to (bio)molecules. Unlike subsequently published applications of the CuAAC for the labeling of molecules with radiometals, their approach utilizes the 1,2,3-triazole not only as a mere linker to connect a molecule with a radiometal chelate (conjugate design), but makes use of the excellent coordination properties of the heterocycle as part of a functional ligand system in an integrated approach [22]. The broad applicability and efficiency of this ^99m^Tc-radiolabeling strategy, in particular those provided by convenient one-pot procedures developed in the course of the work, prompted the authors to term their approach “Click-to-Chelate” [21]. This review summarizes original articles on the Click-to-Chelate concept published since 2006. Examples of applications of the CuAAC in which the 1,2,3-triazole is not part of a chelating system but just tethers a radiometal complex to various molecules are not included. Particular attention has been paid to examples of “clicked” ^99m^Tc-radiotracers which describe biological evaluations *in vitro* and *in vivo*, as this is an important aspect for potential translation of the compounds into the clinic.

## 3. Click-to-Chelate

**Scheme 2:**
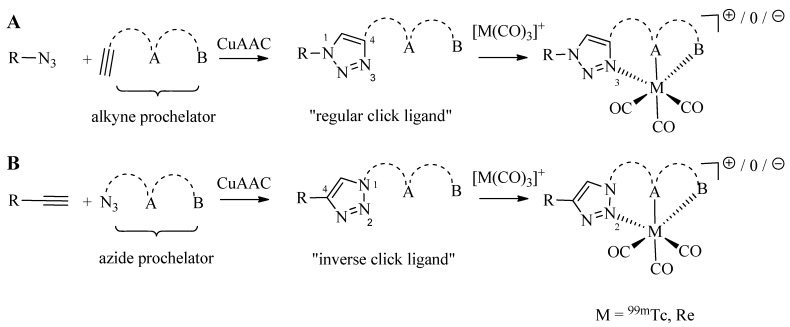
Schematic representation of the synthesis of regular (A) and inverse (B) 1,2,3-triazolyl ligand systems for the complexation of the [M(CO)_3_]^+^ core. A and B represents various functional groups for coordination of the tricarbonyl core.

As discussed in the introduction, the preparation of tridentate chelating systems (and BFCAs thereof) for the complexation of [M(CO)_3_]^+^ generally requires multi-step synthesis and their incorporation into (bio)molecules often lacks efficiency and selectivity due to cross-reactivity with other functional groups present. The chemical orthogonality of the CuAAC together with the ease of the introduction of alkyne and azide moieties into molecules by synthetic or biochemical methods [23,24,25,26,27] provides a solution to these shortcomings. Thus, the reaction of azide-functionalized compounds with alkyne prochelators provides 1,4-disubstituted 1,2,3-triazole-containing tridentate ligand systems (termed “regular click ligands”; Scheme 2A) [21]. Similarly, cycloaddition of alkyne derivatives with azide prochelators yields isomeric triazolyl products (referred to as “inverse click ligands”; Scheme 2B), which are set up to coordinate to [M(CO)_3_]^+^ via the nitrogen in position 2 of the triazole heterocycle, rather than the nitrogen in position 3 of “regular click ligands” (see below). Noteworthy, all ligand systems can be prepared without the use of protective groups in a single step, high yields, and under mild reaction conditions suitable for the functionalization of delicate biomolecules. Reaction of the tridentate chelators with [M(CO)_3_]^+^ gives structurally diverse organometallic complexes that differ in size, lipophilicity, and overall charge (Table 1). All these characteristics are important for fine tuning the physicochemical properties of a radiotracer for its application *in vivo*.

The radiolabeling of regular click ligands (**L**; Table 1, entries 1–5) with [^99m^Tc(CO)_3_]^+^ proceeds smoothly under standard reactions conditions (PBS, pH 7.4, 100 °C, 20–30 min) [21]. Quantitative formation of [^99m^Tc(CO)_3_(**L**)] can be achieved at a ligand concentration in the micromolar range (10^–5^–10^–6^ M), which is comparable to that of histidine (or Nτ-substituted histidines), one of the most efficient chelators for complexation of the ^99m^Tc tricarbonyl core [28]. Particularly for receptor targeting radiopharmaceuticals, it is important that high specific activities can be achieved (e.g., by employing low ligand concentrations) in order to avoid interfering receptor saturation. On the other hand, quantitative radiolabeling of the ^99m^Tc-tricarbonyl core with inverse click ligands (e.g., Table 1, entry 6; for additional examples see ref. [21]) requires a ligand concentration orders of magnitudes higher (10^–3^–10^–2^ M) than that of regular click ligands. This indicates a decreased efficiency of the inverse click ligand systems likely due to the lower electron density at N2 compared to that of N3 of the triazole heterocycle as shown by density functional theory (DFT) calculations [21].

**Scheme 3:**
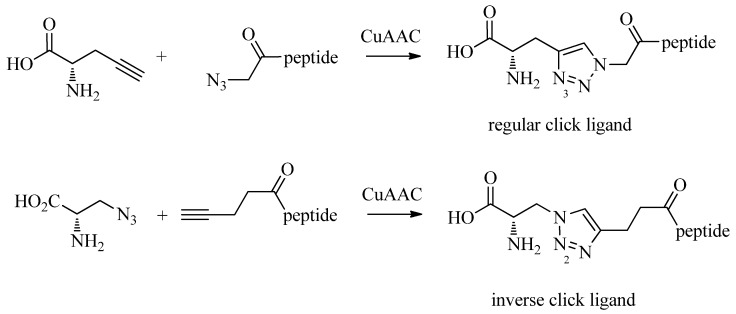
Regular and inverse click ligands conjugated to the N-terminus of a bombesin derivative. Peptide = (ßAla)_1–2_[Cha^13^, Nle^14^]BBS(7–14); the N-terminal ßAla units serve as a spacer between the peptide and the metal chelate.

Brans *et al*. have conjugated both variations of click ligands to the N-terminus of a tumor-targeting bombesin (BBS) derivative (Scheme 3; see below for the relevance of BBS in nuclear oncology) [29]. Radiolabeling of the two peptides with [^99m^Tc(CO)_3_]^+^ under standard conditions revealed a significantly lower radiolabeling yield for the peptide with the inverse click ligand (<60%) in comparison to the compound with the regular click ligand (>95%). Moreover, the ^99m^Tc adduct of the inverse ligand system was found to be unstable and thus, not suitable for *in vivo* evaluation. Anderson *et al*. have reported a similar instability of a ^nat^Re-tricarbonyl based luminescent probe in which the metal is coordinated by the N2 nitrogen of triazole ligands [30]. Even though different metal complexes of 1,4-disubstituted 1,2,3-triazoles with coordination can be synthesized via the N2 [31], the low chelation efficiency combined with the modest stability of the complexes in biological media makes them unlikely to find biological or medical applications. It is therefore important that 1,2,3-triazole-based metallic bioprobes are designed in a way that ensures coordination via the N3 of the heterocycle.

**Table 1:** Examples of prochelators, formation of tridentate triazolyl ligands by CuAAC with benzyl azide (entry 1–5) or 3-phenyl-1-propyne (entry 6), and complexes with [M(CO)_3_]^+^ thereof.

Entry	Prochelator	Tridentate ligand (L) ^1^	Metal complex ^2^
**1** ^3^	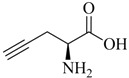	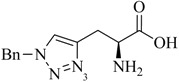	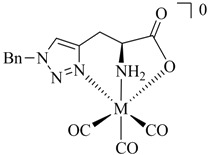
**2** ^4^	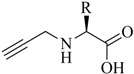	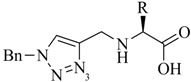	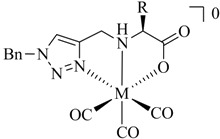
**3**	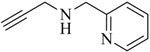	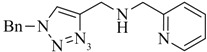	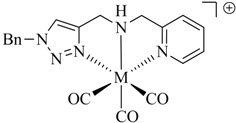
**4**	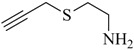	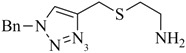	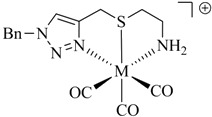
**5**	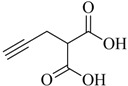	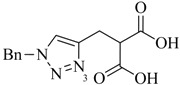	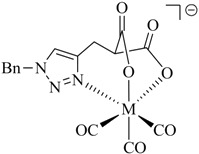
**6** ^3, 5^	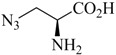	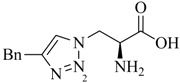	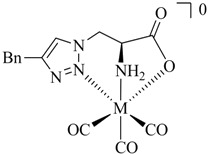

^(1)^ Coordinating nitrogen of the 1,2,3-triazole heterocycle is indicated; ^(2)^ M = ^99m^Tc, ^nat^Re; ^(3)^ commercial compounds; ^(4)^ prochelators derived from amino acids (R = amino acid specific side chain); ^(5)^ example of an inverse click ligand.

**Scheme 4:**
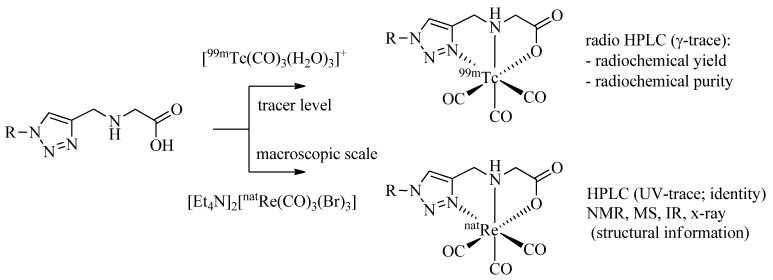
Labeling of a representative example of a regular click ligand with ^99m^Tc- and ^nat^Re-tricarbonyl for identification and characterization of the organometallic products.

**Scheme 5:**
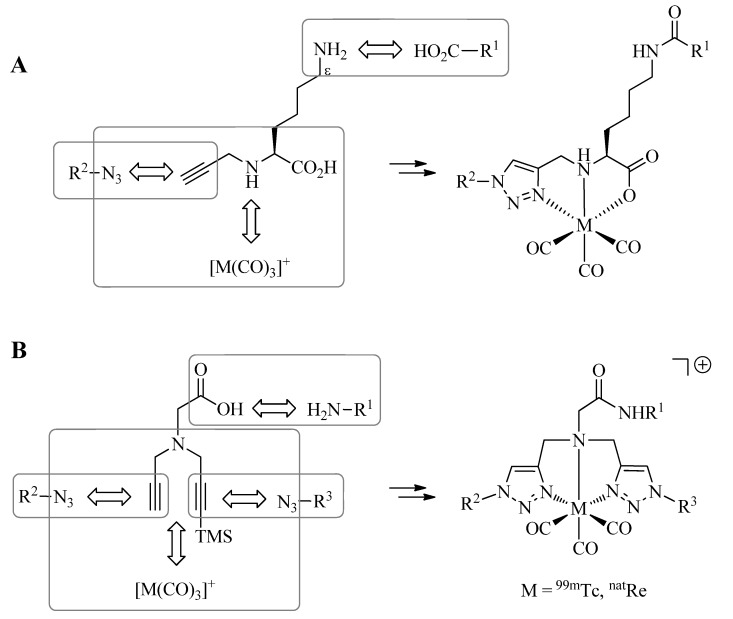
Extension of the Click-to-Chelate approach provides efficiently trifunctional (**A**), or tetrafunctional (**B**) conjugates. TMS = trimethylsilyl group.

It is common practice to identify ^99m^Tc-complexes prepared on a no carrier added (n.c.a.) level by comparison with the corresponding non-radioactive ^nat^Re analogues by HPLC (*γ*-trace *versus* UV-trace; Scheme 4). In addition, the corresponding ^nat^Re-tricarbonyl complexes allow structural analysis by spectrometric and spectroscopic methods including IR, MS, and NMR. ^nat^Re analogues of ^99m^Tc-tricarbonyl complexes can be synthesized conveniently on a macroscopic scale by the reaction of the ligand system of interests with [Et_4_N]_2_[Re(CO)_3_(Br)_3_] [32] in alcohol or water according to published procedures. In all cases discussed above (and more examples have been reported in the meantime), the corresponding ^nat^Re-tricarbonyl complexes were prepared and fully characterized. In addition, more than half a dozen reported X-ray structures confirm the formation of *fac*-M(CO)_3_ complexes by tridentate coordination of the triazolyl ligands as depicted in, e.g., Figure 1, Table 1, or Scheme 5B [33,34,35,36].

**Figure 2:**
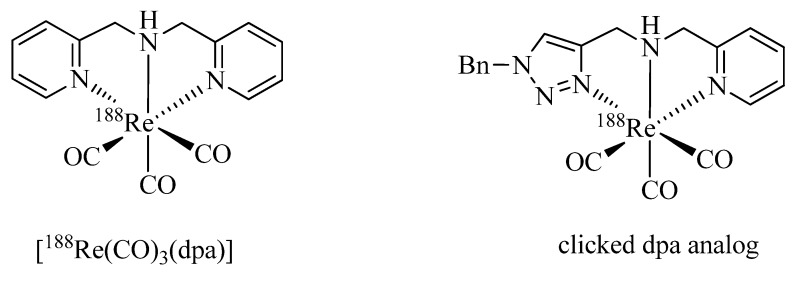
*Fac*-^188^Re-tricarbonyl complexes of dpa and a regular click ligand.

As pointed out above, a large number of non-radioactive ^nat^Re-tricarbonyl complexes have been reported for structural elucidation and identification of the analogous ^99m^Tc complexes. Only recently have Wang *et al*. reported the first example of a chelate in which the ^188^Re-tricarbonyl core is coordinated by a click ligand (Figure 2) [37]. In their work, the efficiency of the complexation of [^188^Re(CO)_3_(H_2_O)_3_]Br with bis(pyridine-2-ylmethyl)amine (dpa) was compared with that of a regular click ligand. Even though dpa was shown to be the more efficient chelator, respectable radiolabeling yields (>80%) were achieved with the regular click ligand system.

## 4. One-Pot Click-to-Chelate Procedures

**Scheme 6:**
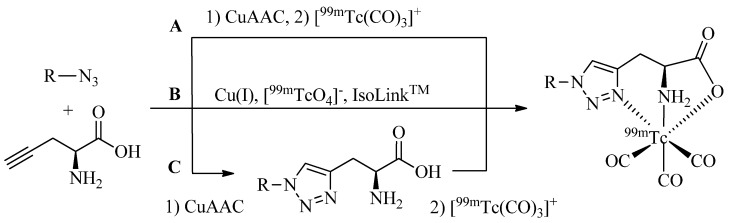
Example of one-pot, two-step (**A**) and one-pot, single-step (**B**) Click-to-Chelate procedures yielding the same radiolabeled compounds as obtained by multi-step synthesis with pre-synthesized and isolated triazolyl ligand systems (**C**).

While the ligands described above, in particularly the regular click ligands, represent a class of excellent chelators for the complexation of [M(CO)_3_]^+^, the individual azide and alkyne substrates as well as the various additives required for CuAAC are poor ligands and do not form stable or defined complexes with the organometallic tricarbonyl core [21]. Exploitation of this observation led to the development of efficient procedures by which the synthesis of the ligand system, its conjugation to a molecule of interest, and the radiolabeling with [M(CO)_3_]^+^ can be achieved in a single pot without isolation and purification of intermediates. Thus, heating of an aqueous solution of azide substrates and alkyne prochelators in the presence of catalytic amounts of Cu(I) for 20–30 min at 100 °C followed by addition of [^99m^Tc(CO)_3_]^+^ and a second heating step (Scheme 6A) provided cleanly the same ^99m^Tc tricarbonyl labeled compounds in identical radiochemical yields and purity as obtained by the reaction of pre-synthesized and isolated triazolyl ligands with [^99m^Tc(CO)_3_]^+^ (Scheme 6C). Remarkably, the same ^99m^Tc-tricarbonyl complexes can be obtained in some instances by simply mixing all substrates with the generator eluent containing pertechnetate ([^99m^TcO_4_]^–^) in the presence of the IsoLink™ kit (Scheme 6B). In the meantime, a number of examples of successful one-pot, two-step- or single-step procedures have been reported for different click-ligand systems (see below). In some cases, the reaction time of individual steps can be accelerated by microwave heating (unpublished results). Simplification of radiolabeling procedures as illustrated by the one-pot examples shown are of fundamental importance for facilitating the translation of novel labeling strategies into routine practice for clinical applications in nuclear medicine.

## 5. Comparison of Click Ligands with Established Chelators for [M(CO)_3_]^+^

**Figure 3:**
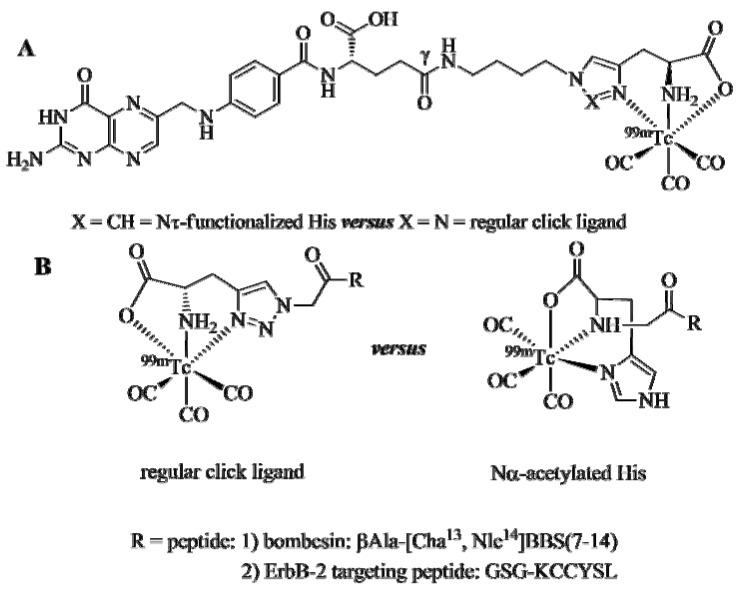
Comparison of regular click ligands with established chelators for the labeling of tumor-seeking vectors with the ^99m^Tctricarbonyl core. (**A**) Folic acid conjugated to a Nτ-derivatized His chelator or a regular click ligand. (**B**) Two peptides functionalized with a NαAc His chelator or a regular click ligand. The ßAla and the GSG motifs are spacer units that separate the receptor targeting peptide from the chelate.

As true for any new ligand system designed for the formation of radiometal complexes for use in nuclear medicine, its performance should be scrutinized and compared with established systems in order to evaluate its general utility. For regular click ligands, this was accomplished by a direct side-by-side comparison *in vitro* and *in vivo* with two gold standard chelators for [^99m^Tc(CO)_3_]^+^, namely Nτ-derivatised histidine (His) and Nα-acetylated His (Figure 3). Towards this goal, Mindt *et al*. [38] Brans *et al*. [29], and Zhang *et al*. [39] have applied the click-to-chelate approach to tumor-targeting vectors of relevance in nuclear oncology and studied the biological characteristics of the ^99m^Tc-labeled conjugates.

Radiolabeled folic acid derivatives functionalized at the γ-position with a radiometal chelate have been shown to be promising candidates for the development of radiotracers specific for folate receptors (FR), which are overexpressed by tumors of the ovary, cervix, endometrium, lung, kidney, breast, colon, and brain [40]. Bombesin (BBS) is a peptide, which targets the gastrin-releasing peptide receptor (GRPr). BBS and derivatives thereof are interesting vectors for the development of tumor targeting agents because of the overexpression of the GRPr by different tumor cells including prostate, breast, colon, and small-cell lung carcinomas [41]. Finally, the peptide KCCYSL, identified by phage display, is a small peptide specific for the ErbB-2 receptor, a member of the epidermal growth factor receptor family, which is overexpressed by breast and prostate carcinoma cells [39].

The comparative studies of the ^99m^Tc-tricarbonyl labeled folate (Figure 3A), BBS, and ErbB-2-targeting peptide derivatives (Figure 3B) clearly demonstrated the equivalence of the clicked ligand system *versus* the established Nτ-derivatized His and Nα-acetylated His chelators. Radiolabeling yields and purities achieved with the conjugates bearing a regular click ligand were equal to that of the conjugates equipped with the reference chelators, as were their blood serum stabilities, cell internalization properties, receptor affinities, and receptor specificities. Most importantly, the nature of the ligand system did not influence the biodistribution of the radioconjugates as determined by experiments *in vivo* with the corresponding mouse models.

Also, Ferro-Flores *et al*. have compared a bombesin derivative, [Lys^3^]BBS(1–14), radiolabeled with [^99m^Tc(CO)_3_]^+^ via a regular click ligand with an analogous compound radiolabeled with technetium(III) using the EDDA/HYNIC (ethylenediamine *N,N*'-diacetic acid/hydrazinonicotinamide) chelating system [42]. As expected, the ^99m^Tc-tricarbonyl radiopeptide exhibited an increased lipophilicity and thus, higher hepatobiliary excretion than the EDDA/HYNIC derivative. The authors conclude that the BBS conjugate radiolabeled with the ^99m^Tc-tricarbonyl core via a regular click ligand is nevertheless the more promising candidate for clinical development because, unlike in the case of HYNIC chelates [43], it consists of a structurally defined metal complex on a tracer level, an important prerequisite for the approval of new radiopharmaceuticals by regulatory authorities. 

It is important to note that Click-to-Chelate not only offers an efficient and convenient way for the introduction of a ligand system into (bio)molecules of interest and their radiolabeling with [^99m^Tc(CO)_3_]^+^ but also reduces significantly the synthetic effort to obtain the corresponding precursors, e.g., alkyne prochelators (or BFCAs with respect to other ligand systems). This benefit of Click-to-Chelate may not be obvious in the case of the modification of peptides to which different entities, including various ligand systems, can conveniently conjugated to on solid support. However, the advantages of Click-to-Chelate become more apparent when applied to the synthetically more challenging situation of the selective modification of polyfunctional molecules in solution. For example, the synthesis of 1,2,3-triazole-modified folic acid (Figure 3A) was achieved by Click-to-Chelate in eight synthetic steps with an overall yield of approx. 80% [38]. In comparison, the analogous ^99m^Tc-tricarbonyl labeled folic acid derivative equipped with an Nτ-derivatized His chelator required ten synthetic steps and provided the final radiotracer in < 1% yield [44]. This example, as well as others discussed below, demonstrates the synthetic efficiency of the Click-to-Chelate strategy for the assembly of ^99m^Tc tricarbonyl radiotracers.

## 6. Multifunctional Radioconjugates by Extended Click-to-Chelate

So far, the synthesis of tridentate 1,4-disubstituted 1,2,3 triazole-containing ligand systems and their simultaneous conjugation to molecules of interest by means of the CuAAC have been discussed. The Click-to-Chelate approach can be readily extended to the preparation of multifunctional ^99m^Tc-tricarbonyl radioconjugates by structural modifications of the prochelators employed. Mindt *et al*. have demonstrated the feasibility of such applications by two different approaches [34,36]. In a first strategy, a lysine-derived prochelator is employed to which various molecules of interest (R^1^) can be selectively conjugated to via the Nε-amine functionality by amide bond formation (Scheme 5A). CuAAC with a second, azide-functionalized molecule (R^2^) covalently links the two molecules via the formed regular click ligand. Thus, the final product combines a nuclear reporter probe (^99m^Tc) with two different chemical (or biological) entities in a trifunctional conjugate. The strategy was successfully applied to the preparation of a ^99m^Tc-labeled conjugate comprising a tumor-targeting peptide sequence ([Cha^13^, Nle^14^]bombesin(7–14)) and a low molecular weight albumin binder, a pharmacological modifier to elongate the blood circulation time of the conjugate. Evaluation of the trifunctional conjugate *in vitro* and *in vivo* provided promising results for its use as a SPECT imaging agent for the visualization of GRPr-positive tumors [34]. Recently, Kluba *et al*. have reported yet another application of this strategy by combining the ^99m^Tc-tricarbonyl core with two moieties, each specific for an extra- and intracellular target, respectively [45]. The trifunctional conjugate was designed to target cancer cells by interaction of a BBS fragment (R^2^) with GRPr and, after internalization, with mitochondria via an organelle-specific triphenylphosphonium moiety (R^1^). Adding a moiety specific for an intracellular target to a tumor-targeting peptide conjugate could improve the cellular retention of radioactivity after its successful delivery to tumors and metastases and thus, improve the efficacy of radiopeptide conjugates.

The second approach to multifunctional conjugates involves chelators containing two 1,4-disubtituted 1,2,3-triazoles derived from the CuAAC of azides with a bis-alkyne precursor (Scheme 5B) [36]. The latter can be readily obtained by the stepwise Nα-alkylation of, e.g., glycine with propargyl bromide or silyl protected derivatives thereof. One molecule (R^1^) can be selectively linked to the glycine precursor by amide bond formation. The other two molecules (R^2^ and R^3^) are conjugated by sequential CuAAC, first with the terminal alkyne and, after removal of the silyl protective group, with the second alkyne functionality. Thus, this strategy provides access to overall tetrafunctional conjugates by joining three different chemical or biochemical moieties with the ^99m^Tc-tricarbonyl core. The utility of the approach was demonstrated by the conjugation of a GRPr targeting bombesin derivative with a related bis-triazole ligand system to which two carboxylates were linked to by the CuAAC [36]. The observed low renal uptake and retention of the conjugate *in vivo* suggests that the two appending carboxylates act as pharmacological modulators, which successfully masked the otherwise unfavorable positive charge of the conjugate as the result of the cationic metal chelate.

An almost infinite number of possible combinations of different chemical or biological moieties with the ^99m^Tc-tricarbonyl core can be envisioned by the extended Click-to-Chelate approach and potential applications to radiopharmaceutical development are galore. For example, the combination of tumor-avid vectors with pharmacological modifiers could lead to the development of radiotracers with improved pharmacological profiles, whereas the use of additional imaging probes or therapeutic agents may lead to the design of multimodal conjugates or theranostics. The exploitation of the strategies described has just begun and it is possible that ongoing research efforts directed towards the combination of different entities with the ^99m^Tc-tricarbonyl core will lead to the development of novel radiopharmaceuticals with improved properties for application in molecular imaging and/or receptor-mediated radionuclide therapy.

## 7. Applications of Click-to-Chelate to Radiotracer Development

In the following section, various reported applications of Click-to-Chelate to the development of radiotracers will be summarized, some examples of which have been mentioned already in the text above (including an example of a vitamin, namely folic acid, vitamin B9) [38]. Compounds reported without preclinical *in vitro* and/or *in vivo* data were not included (e.g., “clicked” phospholipids) [21].

**Figure 4:**
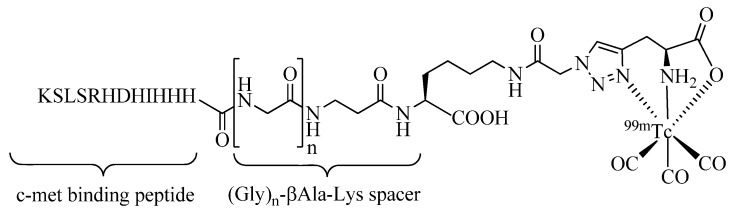
Investigations of the effect of the length of the spacer between a clicked-^99m^Tc-tricarbonyl chelate and the tumor targeting c-met binding peptide.

Click-to-Chelate has been successfully applied to the ^99m^Tc-tricarbonyl labeling of different tumor avid peptides including derivatives of bombesin and the ErbB-2-targeting peptides (see above). In these studies, the general utility of “clicked” triazolyl ligand systems for applications to ^99m^Tc-tricarbonyl labeled peptides and multifunctional radioconjugates was demonstrated. A further example has been reported by Kim *et al*. (Figure 4) who have labeled an epitope-mimicking peptide of the hepatocyte growth factor (HGF)-mesenchymal-epithelial transition factor (Met) molecular pathway, which affects cancer development at different stages [46]. In their work, the length of a spacer unit that separates the c-met binding peptide from the ^99m^Tc-tricarbonyl chelate was investigated. They have shown that the modification of the peptide was readily accomplished by Click-to-Chelate providing radiopeptides of good stability and excellent binding affinity to the c-met receptor. Based on the *in vitro* evaluation of the radiopeptides investigated, a spacer unit consisting of three glycine units (n = 3) provided the most promising results.

**Figure 5:**
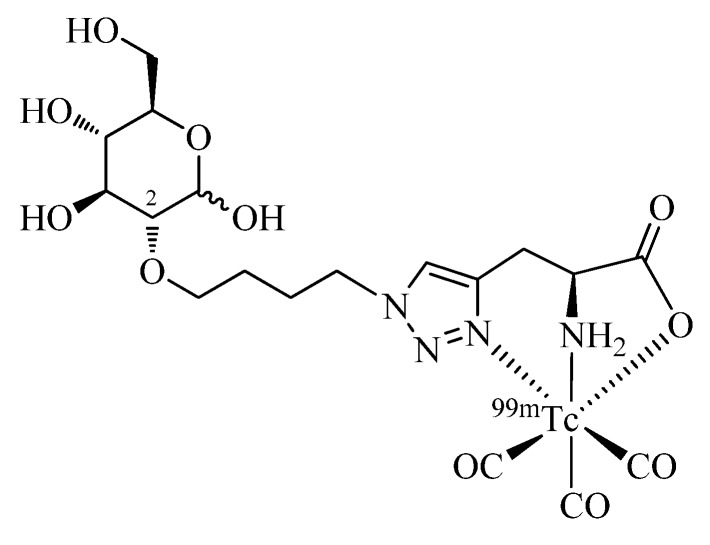
A glucose derivative functionalized at the C2 position with a regular click ligand by the one-pot, 2-step Click-to-Chelate procedure.

Glucose is considered as a key molecule in metabolic evaluation of tumors due to the up-regulation of glycolysis, which results in an increased consumption of the carbohydrate in tumor tissue [47]. In the past, different strategies have been reported for the identification of a ^99m^Tc labeled glucose derivative as a more readily available SPECT surrogate of the established PET tracer [^18^F]-2-fluoro-2-deoxy-D-glucose (FDG) [48]. Fernández *et al*. have prepared a glucose derivative functionalized at the C2 position by the one-pot, 2-step Click-to-Chelate protocol (Figure 5) and studied its biodistribution in comparison to FDG in a lung carcinoma mouse model [49]. Reported tumor uptake *in vivo* of the radiotracer was moderate in comparison to FDG and likely due to passive diffusion of the hydrophilic compound. As in the case of other reported ^99m^Tc/^nat^Re-labeled glucose derivatives, specific uptake via the GLUT-1 receptor pathway could not be confirmed.

**Figure 6:**
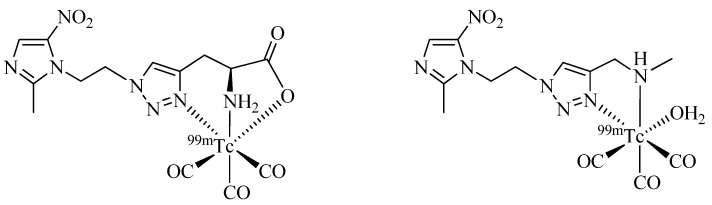
Comparison of tri- and bidentate click chelators applied to the development of novel hypoxia radiotracers based on 5-nitro-imidazoles.

Click-to-Chelate has also been employed to the development of hypoxia imaging agents, which are important radiotracers for the assessment of the oxygenation status of tumors critical for determining the effectiveness of radiation therapy [50]. Fernández *et al*. have investigated the utility of bi- and tridentate click chelators for the ^99m^Tc-tricarbonyl labeling of 5-nitro-imidazole derivatives prepared by one-pot Click-to-Chelate protocols (Figure 6) [51]. Evaluation of the compounds *in vitro* and *in vivo* revealed that the compound equipped with a tridentate chelator was superior in terms of blood serum stability, plasma protein binding, and tumor uptake. In addition, the “clicked” 5-nitro-imidazole outperformed other ^99m^Tc-labeled hypoxia imaging agents previously investigated by the same group (e.g., 5-nitroimidazol labeled with ^99m^Tc(V) nitrido- or ^99m^Tc(III) [4+1] chelates). The authors conclude that the compound with a tridentate regular click ligand is so far their most promising candidate for the development of a ^99m^Tc radiopharmaceutical for hypoxia tumor diagnosis.

**Scheme 7:**
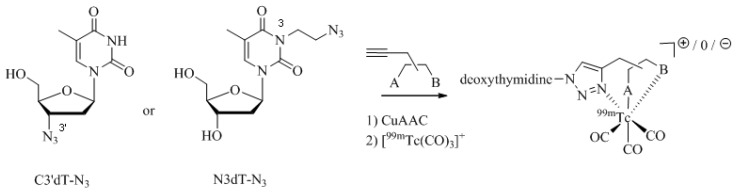
Synthesis of thymidine analogs functionalized with regular click ligands at the C3' or N3 position developed as substrates for hTK1. dT = deoxythymidine; A and B represent functional groups for coordination of the ^99m^Tc-ricarbonyl core.

Analogs of the nucleoside thymidine have the potential to be recognized as substrates by the human cytosolic thymidine kinase (hTK1). Since hTK1 is overexpressed in a wide variety of cancer cells, radiolabeled thymidine derivatives represent potential markers for cancer cell proliferation. Struthers *et al*. have reported the synthesis and *in vitro* evaluation of ^99m^Tc-tricarbonyl labeled thymidine analogs in an effort to develop a radiotracer for SPECT more readily available compared to the currently used PET tracer 3-deoxy-3'-[^18^F]fluorothymidine (FLT) [35,52]. They prepared a set of ^99m^Tc-tricarbonyl labeled thymidine derivatives in a combinatorial fashion by parallel one-pot Click-to-Chelate synthesis (Scheme 7; see Table 1, entry 1–5 for examples of alkyne precursors employed). The products obtained differ not only in the position of functionalization (C3' *versus* N3) but also with regards to the size and overall charge of the radiometal chelate. Substrate activity studies carried out by phosphorylation assays showed that substrate recognition by hTK1 is dependent on both, the site of functionalization of thymidine as well as the charge of the ^99m^Tccomplex. In general, C3'-functionalized compounds were the better substrates for hTK1 and a preference for neutral or anionic complexes was demonstrated. This elegant work has led to the identification of the first metal containing hTK1substrates.

**Figure 7:**
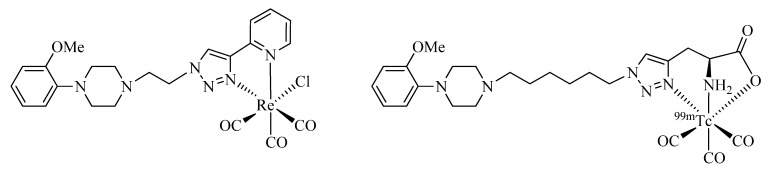
Bi- and tridentate clicked ligands conjugated to MPP for the development of CNS imaging agents.

Seridi *et al*. [53] and Hassanzadeh *et al*. [54,55] have used CuAAC in the development of brain radiotracers. They describe the conjugation of bi- and tridentate regular click ligands to 1-(2-methoxyphenyl)piperazine (MPP), a pharmacophore frequently found in imaging agents selective for 5-HT_1A_, a receptor subtype for the neurotransmitter serotonin (5-hydroxytryptamine, 5-HT; Figure 7). The radiotracers exhibited favorable low protein binding, good blood serum stability, and a lipophilicity suitable to cross the blood-brain barrier (BBB). The compound functionalized with a tridentate click ligand was labeled with [^99m^Tc(CO)_3_]^+^ and evaluated *in vitro* and *in vivo*. Receptor saturation experiments performed with homogenated hippocampus membranes of rats revealed a high receptor affinity (K_D_) of the compound in the single-digit nanomolar range. *In vivo* experiments with rats indicated a specific uptake of the radiotracer in the brain, being highest in the hippocampus. Both groups conclude that the ^99m^Tc-labeled MPP derivatives have suitable characteristics for use as central nervous system (CNS) receptor specific imaging agents.

**Figure 8:**
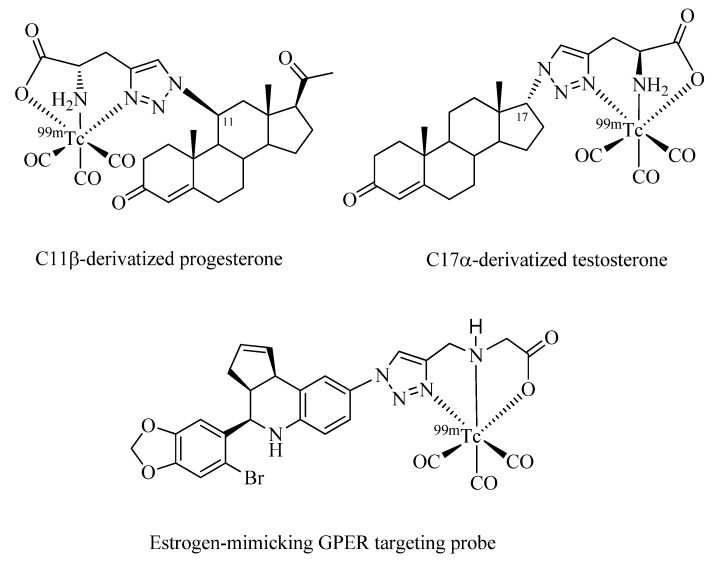
Examples of steroids or analogs thereof radiolabeled with the ^99m^Tc-tricarbonyl core by Click-to-Chelate.

Finally, implementation of Click-to-Chelate to steroid-based radiotracers is discussed (Figure 8). Dhyani *et al*. have investigated ^99m^Tc-tricarbonyl-labeled derivatives of 11ß-progesterone and 17α-testosterone for targeting progesterone (PR) and androgen receptors (AR), which are overexpressed in breast and prostate cancer, respectively [56,57]. The compatibility of the site of functionalization of the steroids with regards to biological function as well as stereochemical considerations were taken into account for the design of the radiotracers. While the synthesis of the compounds was straight forward and the stability of the radiometal complexes in biological media was excellent, experiments *in vivo* using Swiss mice or Wistar rats did not reveal specificities towards the corresponding receptors. These findings may be the result of the bulkiness of the ^99m^Tc chelate in comparison to the steroid vector, which could interfere with the interaction of the compound with its binding site.

Burai *et al*. have reported the ^99m^Tc-tricarbonyl labeling of tetrahydro-*3H*-cyclopenta[c]quinoline, the structural design of which was inspired by estrogen steroids (Figure 8) [58]. Derivatives of the parent compound are specific agonists/antagonists of the recently identified G protein-coupled estrogen receptor (GPER), but not the estrogen ERα/ß receptors. GPERs are involved in tumor signaling pathways and have shown promising properties as putatively important biomarkers or therapeutic targets in oncology. Again, receptor specificity of the vector was lost upon functionalization with the radiometal chelate as shown by *in vitro* assays measuring intracellular calcium immobilization or PI3K activation. In the same work, a series of other ^99m^Tc-labeled derivatives of the parent compound was investigated of which some, particularly those with a spacer moiety between the radiometal chelate and the vector, showed retained activities as GPER agonists/antagonists. Surprisingly, in none of the “clicked” steroid examples discussed above， the effect of a spacer unit between the receptor specific vector and the radiometal chelate has been investigated.

## 8. Summary and Perspectives

Since first reported in 2006, the Click-to-Chelate approach has found numerous applications for the development of radiopharmaceuticals based on the ^99m^Tc-tricarbonyl core. Examples include organometallic radiotracers with potential for applications in nuclear medicine ranging from peptides, vitamins, carbohydrates, steroids to hypoxia imaging agents, substrates for tumor-associated enzymes (e.g. hTK1), and CNS imaging probes. Thus, Click-to-Chelate has had a demonstrated impact on the community involved in radiopharmaceutical sciences. 

The majority of applications discussed in this review employ propargyl glycine (Table 1, entry 1) as an alkyne prochelator. This is likely due to its commercial availability and demonstrated equivalency of the resulting tridentate triazolyl ligand system to Nτ-functionalized His. However, other alkyne derivatives (e.g., readily accessible Nα-propargylated amino acid derivatives; Table 1, entry 2) also hold great promise for future applications, especially in terms of the development of multifunctional radioconjugates.

Meanwhile, new and innovative approaches have been described, which expand the portfolio of click chemistry applications in radiopharmaceutical sciences and eventually in nuclear medicine. With respect to radionuclides of technetium and rhenium, the CuAAC has been employed to BFCAs suitable for the complexation of oxo-technetium species [59], the development of a “click-to-cyclize and chelate” strategy for the cyclization of peptides and their radiolabeling [60], and pre-labeling strategies [61] for the labeling of molecules with pre-formed radiometal complexes. The CuAAC technique has also been applied to the preparation of multiple imaging probes derived from the same precursor for a direct comparison of different imaging probes as well imaging modalities [62]. In addition, copper-free click reactions [63] have become increasingly popular for the development of radiotracers [64,65,66,67,68,69] including examples of pre-targeting approaches [70]. 

Click chemistry, in particular the CuAAC reaction, has demonstrated its high potential for the development of radiotracers. However, there are ample opportunities left for new innovations to be reported in the future and first clinical applications of “clicked” probes are being awaited. Among the many potential applications of click reactions in radiopharmaceutical sciences, Click-to-Chelate will continue to play an important role for the radiolabeling of molecules with the technetium- and rhenium-tricarbonyl cores.
